# Incidence of injection pain between ciprofol and propofol after induction of general anesthesia: a systematic review and meta-analysis of randomized controlled trials

**DOI:** 10.3389/fmed.2026.1749156

**Published:** 2026-02-03

**Authors:** Jinfang Zeng, Guowei Zhu, Jinjin Jian, Minmin Zhu

**Affiliations:** 1 Department of Anesthesiology and Pain Medicine, Jiangnan University Medical Center, Wuxi No. 2 People’s Hospital, Affiliated Wuxi Clinical College of Nantong University, Wuxi, China; 2 Wuxi School of Medicine, Jiangnan University, Wuxi, China; 3 Department of Anesthesiology, Affiliated Hospital of Jiangnan University, Wuxi, China

**Keywords:** ciprofol, injection pain, meta-analysis, propofol, review

## Abstract

**Background:**

Propofol and ciprofol are commonly used agents for inducing general anesthesia. Although propofol is preferred for its potent sedative effects and quick recovery, it is associated with a higher incidence of injection-related pain. In contrast, ciprofol may help alleviate this pain. However, evidence regarding its advantage in reducing injection pain remains insufficient. This systematic review aimed to assess the frequency of pain during administration tied to the use of ciprofol as well as propofol.

**Methods:**

A systematic search of PubMed, Embase, and the Cochrane Central Register of Controlled Trials was conducted to identify randomized controlled trials comparing ciprofol with propofol for the induction of general anesthesia. Two reviewers independently screened studies and extracted data. Meta-analyses were performed using Review Manager software.

**Results:**

Ten trials with a total of 1,402 patients were studied in this meta-analysis. Research results showed that ciprofol markedly reduced the occurrence of pain from injections when contrasted with propofol, exhibiting a risk ratio (RR) of 0.15 (95% confidence interval [CI]: 0.11 to 0.20). Ciprofol significantly reduced the incidence of injection pain when the patient was elderly (RR = 0.14, 95% CI: 0.06 to 0.35), and also non-elderly (RR = 0.15, 95% CI: 0.11 to 0.21). In addition, ciprofol significantly reduced the incidence hypotension (RR = 0.63, 95% CI: 0.46 to 0.87). A trend toward lower tachycardia was observed with ciprofol, but this did not reach statistical significance (RR = 0.63, 95% CI 0.39–1.01). The GRADE assessment revealed moderate certainty regarding the outcomes associated with injection pain. Trial sequential analysis (TSA) suggested that the cumulative evidence for injection pain and hypotension was sufficient and that further trials are unlikely to change these conclusions.

**Conclusion:**

The study revealed that ciprofol significantly decreases the occurrence of injection-related discomfort and was associated with a reduced incidence of hypotension, while a trend toward lower tachycardia was observed. Trial sequential analysis further supported the robustness of these findings.

**Systematic review registration:**

CRD420251003081.

## 1 Introduction

Injection pain during anesthesia can cause significant discomfort, muscle tension, and stress, raising blood pressure and heart rate, especially risky for cardiovascular patients (1). Repeated painful injections may lead to needle phobia, discouraging patients from seeking necessary care. This pain can also result in local inflammation, delayed healing, or infection, impacting recovery and treatment adherence (2). Pain linked to anesthesia may increase a patient’s nervousness and diminish their contentment with healthcare treatments. Improved techniques and local anesthetics can help minimize pain, enhance comfort, and support better health outcomes (3).

Propofol is a commonly utilized intravenous anesthetic, appreciated for its quick onset of action and smooth recovery profile, which makes it ideal for use in surgeries and procedural sedation. However, a significant limitation is its high incidence of injection pain (4). The research by Yuan et al. (5) revealed that up to 51% of patients in the propofol cohort experienced pain during injection. This discomfort not only affects patient experience but also requires additional management, particularly in high-risk or elderly populations. While propofol is known for its efficacy, it can lead to injection pain and poses potential risks such as hypotension and respiratory depression, underscoring the need for alternative agents with fewer side effects and better patient tolerability (6–8). It is worth mentioning that there has been a search for sedatives with better sedation and fewer side effects, especially in elderly and high-risk patients. Ciprofol is a new intravenous anesthetic that operates similarly to propofol by targeting GABA receptors to enhance inhibitory neurotransmission and induce sedation (9, 10). The latest research within the previous 24 months indicates that ciprofol results in considerably less injection discomfort compared to propofol, implying that ciprofol may be an appropriate substitute for propofol (11–13). This reduction is due to ciprofol’s modified chemical structure, which is less likely to irritate the tissue at the injection site (14).

To our knowledge, there has yet to be a quantitative assessment comparing the incidence of injection pain between ciprofol and propofol during general anesthesia. This meta-analysis seeks to analyze and compare the occurrence of injection pain linked to both ciprofol and propofol, offering valuable insights into their respective safety profiles and clinical applicability in anesthesia induction.

## 2 Methods

Ethical approval was not required for this study, as it is a meta-analysis.

### 2.1 Study registration

We conducted a meta-analysis to assess the incidence of injection pain related to ciprofol and propofol administered during general anesthesia, adhering to the PRISMA guidelines. This research has been cataloged under the identifier CRD420251003081 in the PROSPERO database.

### 2.2 Search approach and eligibility standards

A comprehensive and systematic literature search was conducted independently by two reviewers (Z. J. F. and J. J. J.) in the following electronic databases: PubMed, Embase, and the Cochrane Central Register of Controlled Trials (CENTRAL), from database inception to 29 July 2025, without language restrictions. In addition, clinical trial registries (ClinicalTrials.gov and the Chinese Clinical Trial Registry [ChiCTR]) were searched to identify ongoing or unpublished trials. The search strategy combined controlled vocabulary (MeSH terms in PubMed and Emtree terms in Embase) and free-text keywords related to ciprofol, propofol, and injection pain. The main search terms included: (“ciprofol” OR “HSK3486”) AND (“propofol”) AND (“injection pain” OR “pain on injection” OR “vascular pain” OR “infusion pain”) AND (“randomized controlled trial” OR “randomised” OR “RCT”). Reference lists of all eligible studies and relevant reviews were manually screened to identify additional potentially eligible trials. The full electronic search strategies for each database are provided in Supplementary Table S1 to ensure reproducibility.

#### 2.2.1 Research selection

**Figure 1:**
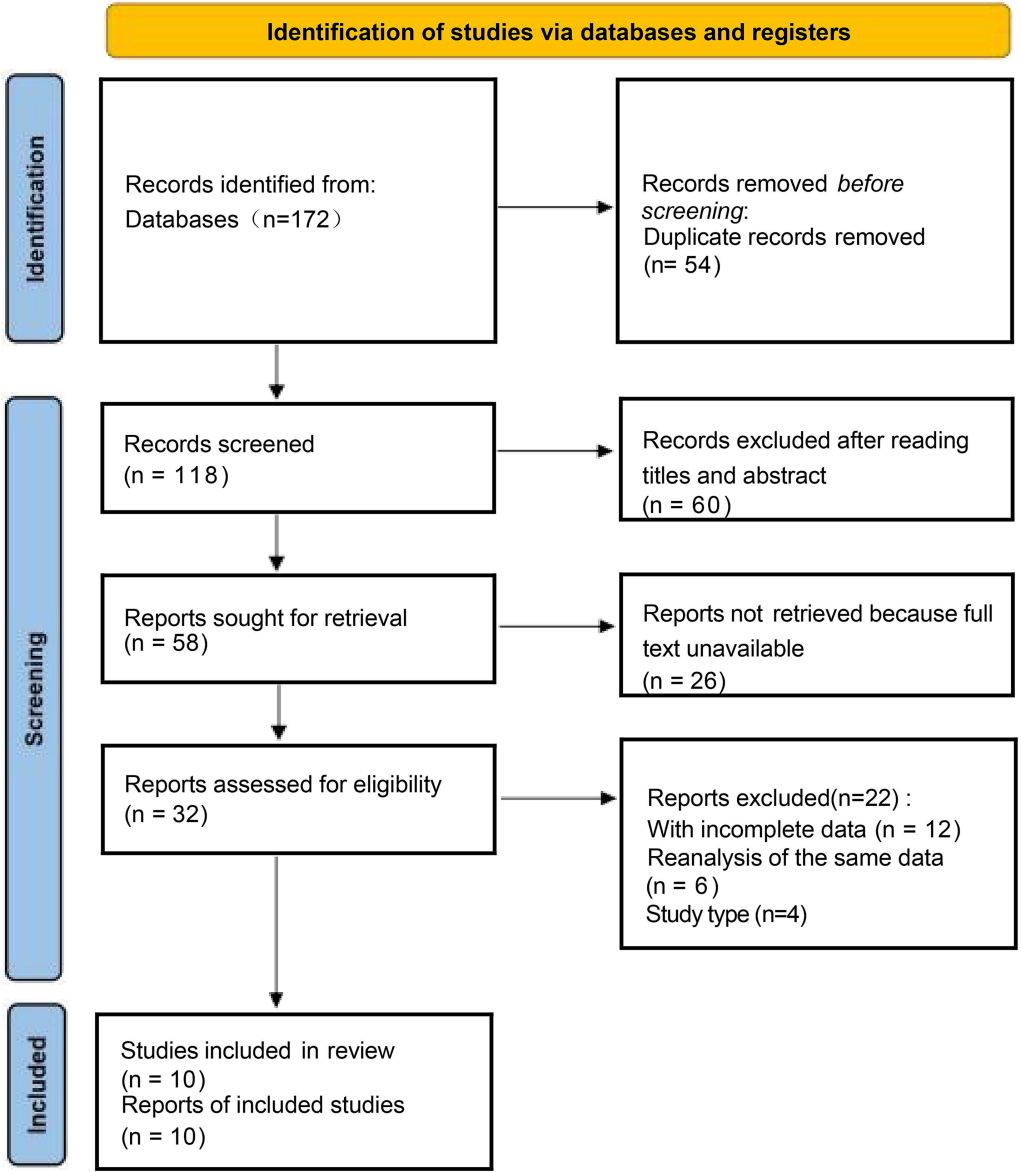
Flow diagram of the inclusion and exclusion process.

Study selection and data extraction were performed in accordance with PRISMA guidelines. Two reviewers (Z. M. M. and Z. G. W.) independently screened all retrieved records in two stages. In the first stage, titles and abstracts were screened to exclude clearly irrelevant studies. In the second stage, full texts of potentially eligible articles were assessed against the predefined inclusion and exclusion criteria. Any disagreement between the two reviewers was resolved through discussion; if consensus could not be reached, a third senior reviewer (Z. M. M.) was consulted for arbitration. Reasons for exclusion at the full-text stage were recorded and are presented in the PRISMA flow diagram (Figure 1). Data extraction was conducted independently by two reviewers (J. J. J. and Z. J. F.) using a standardized data collection form. Extracted items included study characteristics, patient demographics, intervention details, outcome data, and risk-of-bias information. The two extracted datasets were cross-checked, and discrepancies were resolved by consensus or consultation with a third reviewer (Z. M. M.).

#### 2.2.2 Inclusion criteria

Based on the established eligibility criteria, the studies included in this meta-analysis featured the following components:(1) Population: The subjects were patients of both genders with a body mass index (BMI) ranging from 18 to 30 kg/m^2^, all undergoing surgical procedures requiring general anesthesia. (2) Intervention: The analysis was centered on the administration of ciprofol as the primary intervention. (3) Comparator: The studies compared ciprofol to propofol alone, excluding articles that involved a control group comparing ciprofol to other anesthetics. (4) Primary Outcomes: The study measured the occurrence of pain from injections when using ciprofol versus propofol as the principal outcome. Secondary outcomes examined included the frequency of hypotension, hypertension, bradycardia, tachycardia, and postoperative nausea and vomiting (PONV). (5) Study Design: The meta-analysis strictly included solely randomized controlled trials (RCTs) to guarantee the most robust evidence quality for the outcomes. Adhering to these parameters was essential to preserve the stringency and pertinence of the study.

#### 2.2.3 Exclusion criteria

Studies were excluded if they met any of the following criteria:

1. Non-randomized study design (e.g., observational studies, case–control studies, cohort studies, case series, or case reports);
2. Absence of a direct comparison between ciprofol and propofol as the intervention and comparator;
3. Failure to report injection pain;
4. Insufficient or incomplete data that precluded extraction of effect estimates for meta-analysis;
5. Duplicate publications or studies reporting overlapping populations, in which case only the most complete or most recent report was included;
6. Conference abstracts, editorials, narrative reviews, meta-analyses, animal studies, or *in vitro* studies;
7. Studies not conducted in the context of general anesthesia.

These predefined exclusion criteria were applied to ensure methodological rigor and reproducibility.

### 2.3 Information extraction and evaluation of bias risk

Risk of bias was assessed independently by two reviewers (J. J. J. and Z. J. F.) using the Cochrane Risk of Bias tool, which evaluates the following domains: random sequence generation, allocation concealment, blinding of participants and personnel, blinding of outcome assessment, incomplete outcome data, selective reporting, and other sources of bias. Each domain was judged as having low, unclear, or high risk of bias according to the criteria described in the Cochrane Handbook. An overall risk-of-bias judgment for each study was derived based on these domain-level assessments. Disagreements were resolved by discussion or by consultation with a third reviewer (Z. M. M.).

### 2.4 Quality analysis of evidence

We use the GRADE system to ensure the integrity of evidence. It assesses the quality of research based on systematic bias, random errors, and reliability. Evidence is classified into four quality tiers-very low to high-considering factors like bias susceptibility, variability, precision, publication bias, and treatment significance.

### 2.5 Trial sequential analysis

TSA was performed to evaluate whether the cumulative evidence was sufficient and to control the risks of random errors due to sparse data and repeated significance testing. TSA was conducted only for injection pain (primary outcome) and hypotension (key secondary outcome), as these were the outcomes with sufficient numbers of trials and clinical relevance.

TSA was performed using TSA software version 0.9.5.10 Beta. The required information size (RIS) was calculated based on a two-sided type I error (*α*) of 5% and a type II error (*β*) of 20% (power = 80%). The control event proportion was derived from the pooled control group data, and the anticipated intervention effect was based on the relative risk reduction observed in the conventional meta-analysis.

To account for between-trial heterogeneity, the RIS was adjusted using diversity (D^2^). Trial sequential monitoring boundaries for benefit, harm, and futility were constructed accordingly. Evidence was considered sufficient when the cumulative Z-curve crossed the trial sequential monitoring boundary or reached the required information size.

### 2.6 Outcome measures

The study conducted a thorough assessment of injection pain along with the incidence rates of various cardiovascular and postoperative complications, including hypotension, hypertension, bradycardia, tachycardia, and postoperative nausea and vomiting (PONV). This comparison was made between two anesthetic agents: ciprofol and propofol. The analysis utilized pooled relative risk (RR) calculations and 95% confidence intervals (CIs) to quantify the outcomes effectively. To evaluate the overall effect, a Z test was performed, considering a significance level of *p* < 0.05. For studies exhibiting low heterogeneity (*I*^2^ ≤ 50%), a fixed effects model was employed, while a random effects model was adopted for those with higher heterogeneity. Additionally, sensitivity analyses concentrated on studies with low risk of bias, and subgroup analyses were stratified according to the patient’s age.

For each outcome, the number of included studies, total sample size, and event counts were verified and consistently reported across the text, figures, and evidence summaries.

For all binary outcomes, effect estimates were expressed as RRs with 95% confidence intervals to ensure consistency and clinical interpretability.

Procedural characteristics related to injection pain (including injection site, injection rate, cannula characteristics, pre-treatment, and pain assessment methods) were extracted at the study level and are summarized in Supplementary Table S2.

## 3 Results

### 3.1 Study selection

A detailed search of PubMed, Embase, and Cochrane Library identified 172 articles (Figure 1). Initially, 60 studies were excluded for not being controlled trials, followed by 54 due to duplication. An additional 26 studies were removed for not meeting inclusion criteria. After reviewing the remaining 32 articles, 22 were excluded for lacking relevant endpoints. Ultimately, 10 trials (11–13, 15–21) that met the selection criteria were included in the meta-analysis.

### 3.2 Study characteristic

In this meta-analysis, 10 trials (11–13, 15–21) focused on the incidence of injection pain linked to ciprofol and propofol (Table 1). All included studies were published in 2022 or later, predominantly involving adult participants, although a minority of studies also enrolled children. The ciprofol induction doses used in the included trials ranged from 0.2 to 0.5 mg/kg. Surgical procedures assessed included gynecological surgeries, burn eschar excisions, skin grafting, hip fracture operations, and kidney transplantation.

**Table 1:** General information of patients with incidence of injection pain.

Authors	Year	Age	Sex (Male/Female)	BMI, kg m^−2^	Comparisons (group)	Operation	Injection pain	Total
Chen et al. (15)	2022	18 ~ 60 years	0/60	22.2 ± 3.2	Ciprofol 0.4 mg/kg	Gynecological surgery	10	60
			0/60	21.4 ± 2.8	Propofol 2 mg/kg		35	60
Chen et al. (11)	2024	18 ~ 59 years	73/38	-	Ciprofol 0.4 mg/kg	Burn scab excision and skin grafting surgery	4	111
			66/39	-	Propofol 2 mg/kg		31	105
Gan et al. (16)	2024	≥ 18 years	49/119	-	Ciprofol 0.4 mg/kg	Elective surgery with endotracheal intubation	11	168
			26/57	-	Propofol 2 mg/kg		36	83
Lan et al. (12)	2023	18 ~ 70 years	0/75	23.0 ± 2.6	Ciprofol 0.4 mg/kg maintenance 0.6–1.2 mg/kg/h	Hysteroscopy	0	75
			0/74	23.6 ± 2.8	Propofol 2 mg/kg maintenance 3.0–6.0 mg/kg/h.		20	74
Liang et al. (17)	2024	≥ 65 years	34/38	23.6 ± 3.0	Ciprofol 0.2–0.5 mg/kg maintenance 0.4–3 mg/kg/h	Laparoscopic major abdominal surgery	2	72
			40/32	23.6 ± 2.6	Propofol 1–2 mg/kg maintenance 4–12 mg/kg/h		20	72
Lu et al. (18)	2024	≥ 75 years	10/20	-	Ciprofol 0.3 mg/kg	Hip Fracture Surgery	3	30
			14/16	-	Propofol 1.5 mg/kg		15	30
Qin et al. (13)	2022	18 ~ 65 years	18/34	23.38 ± 3.33	Ciprofol 0.4 mg/kg maintenance 0.8–2.4 mg/kg/h	Kidney transplantation	1	52
			18/35	22.63 ± 2.38	Propofol 2 mg/kg maintenance 4–12 mg/kg/h		32	53
Shili et al. (19)	2023	<18 years	25/8	-	Ciprofol 0.4 mg/kg maintenance 1–3 mg/kg/h	Tethered cord surgery	0	33
			19/14	-	Propofol 2 mg/kg maintenance 4–12 mg/kg/h		5	28
Wang et al. (20)	2022	18 ~ 64 years	32/56	23.3 ± 2.9	Ciprofol 0.4 mg/kg	Elective surgery	6	88
			31/57	23.3 ± 3.1	Propofol 2 mg/kg		18	88
Zhu et al. (21)	2024	18 ~ 70 years	20/40	-	Ciprofol 0.4 mg/kg maintenance 0.3–2.4 mg/kg/h	Elective microvascular decompression surgery	2	60
			24/36	-	Propofol 2 mg/kg maintenance 3–12 mg/kg/h		19	60

### 3.3 The methodological quality of the included studies

**Figure 2:**
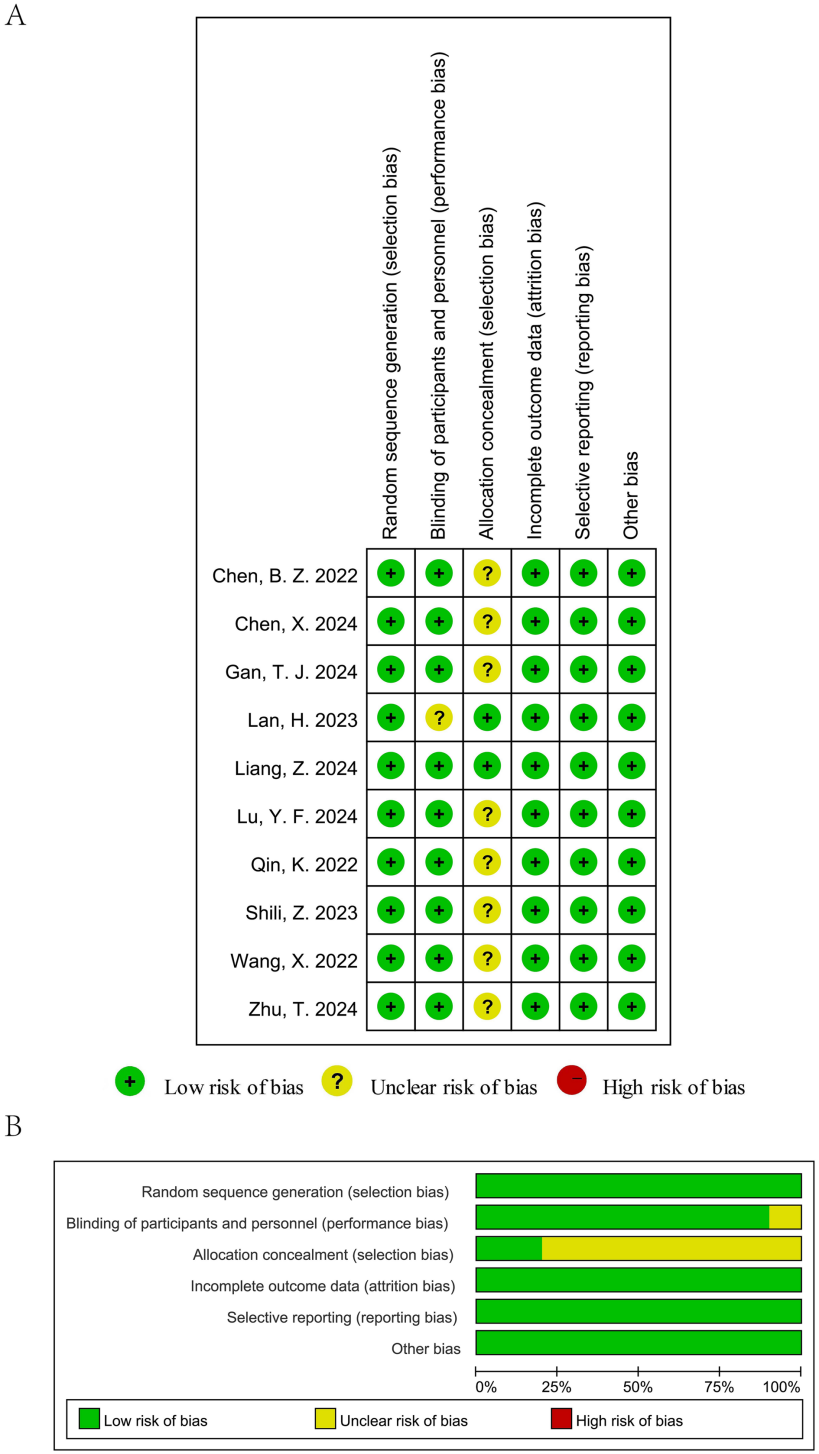
Risk of bias assessment of the included studies: **(A)** Risk of bias summary and **(B)** Risk of bias graph.

Most included trials reported appropriate random sequence generation, indicating a generally low risk of selection bias. However, only two studies explicitly described adequate allocation concealment, while the remaining trials provided insufficient information, resulting in an unclear risk of bias for this domain. Blinding of participants and personnel was reported in several trials, but blinding of outcome assessment was often not clearly described, leading to an unclear risk of detection bias in a proportion of studies. Attrition bias was judged to be low across all trials, as outcome data were complete or nearly complete. No evidence of selective outcome reporting was identified. Overall, while most studies were randomized and had low risk of attrition and reporting bias, several domains—particularly allocation concealment and blinding—were rated as unclear, which may have introduced some risk of bias. The detailed risk-of-bias judgments are presented in Figure 2.

### 3.4 Quality of evidence

The GRADE system showed moderate-certainty evidence for injection pain and hypotension, mainly downgraded for imprecision (total events <300) or inconsistency (*I*^2^ > 50%), while bradycardia was rated as low certainty due to both inconsistency and imprecision (Table 2).

**Table 2:** Grade summary of efficacy of ciprofol and propofol.

Outcome	No. of studies (RCTs)	Participants (ciprofol / propofol)	Relative effect (RR, 95% CI)	Absolute effect*	Certainty of evidence (GRADE)	Rationale for downgrading/upgrading
Injection pain	10	749/653	0.15 (0.11–0.20)	301 fewer per 1,000 (from 283 to 315 fewer)	⊕ ⊕ ⊕◯ Moderate	Downgraded one level for imprecision, as the total number of events was <300, despite a large and consistent effect.
Hypotension	8	656/565	0.63 (0.46–0.87)	141 fewer per 1,000 (from 87 to 185 fewer)	⊕ ⊕ ⊕◯ Moderate	Downgraded one level for inconsistency due to substantial heterogeneity among studies (*I*^2^ > 50%).
Bradycardia	8	548/542	0.94 (0.58–1.52)	1 fewer per 1,000 (from 37 fewer to 46 more)	⊕ ⊕ ◯◯ Low	Downgraded for inconsistency (*I*^2^ > 50%) and imprecision, as the confidence interval was wide and crossed the line of no effect.
Tachycardia	5	305/305	0.63 (0.39–1.01)	48 fewer per 1,000 (from 79 fewer to 0 more)	⊕ ⊕ ⊕◯ Moderate	Downgraded one level for imprecision, as the confidence interval included the possibility of no effect.
Hypertension	6	493/407	0.81 (0.50–1.31)	15 fewer per 1,000 (from 40 fewer to 25 more)	⊕ ⊕ ⊕ ⊕ High	Not downgraded: low risk of bias, consistent results, direct evidence, and acceptable precision. No upgrading applied as the effect was not statistically significant.
PONV	5	440/356	1.06 (0.56–2.00)	3 more per 1,000 (from 20 fewer to 46 more)	⊕ ⊕ ⊕◯ Moderate	Downgraded one level for imprecision, owing to wide confidence intervals crossing the line of no effect.

RR, risk ratio; CI, confidence interval; RCT, randomized controlled trial.

GRADE Working Group grades of evidence: High certainty (⊕ ⊕ ⊕⊕): very confident that the true effect lies close to that of the estimate. Moderate certainty (⊕ ⊕ ⊕◯): moderately confident; the true effect is likely close to the estimate, but may be substantially different. Low certainty (⊕ ⊕ ◯◯): limited confidence.

### 3.5 Results of meta-analysis

#### 3.5.1 Ciprofol versus propofol on injection pain

**Figure 3:**
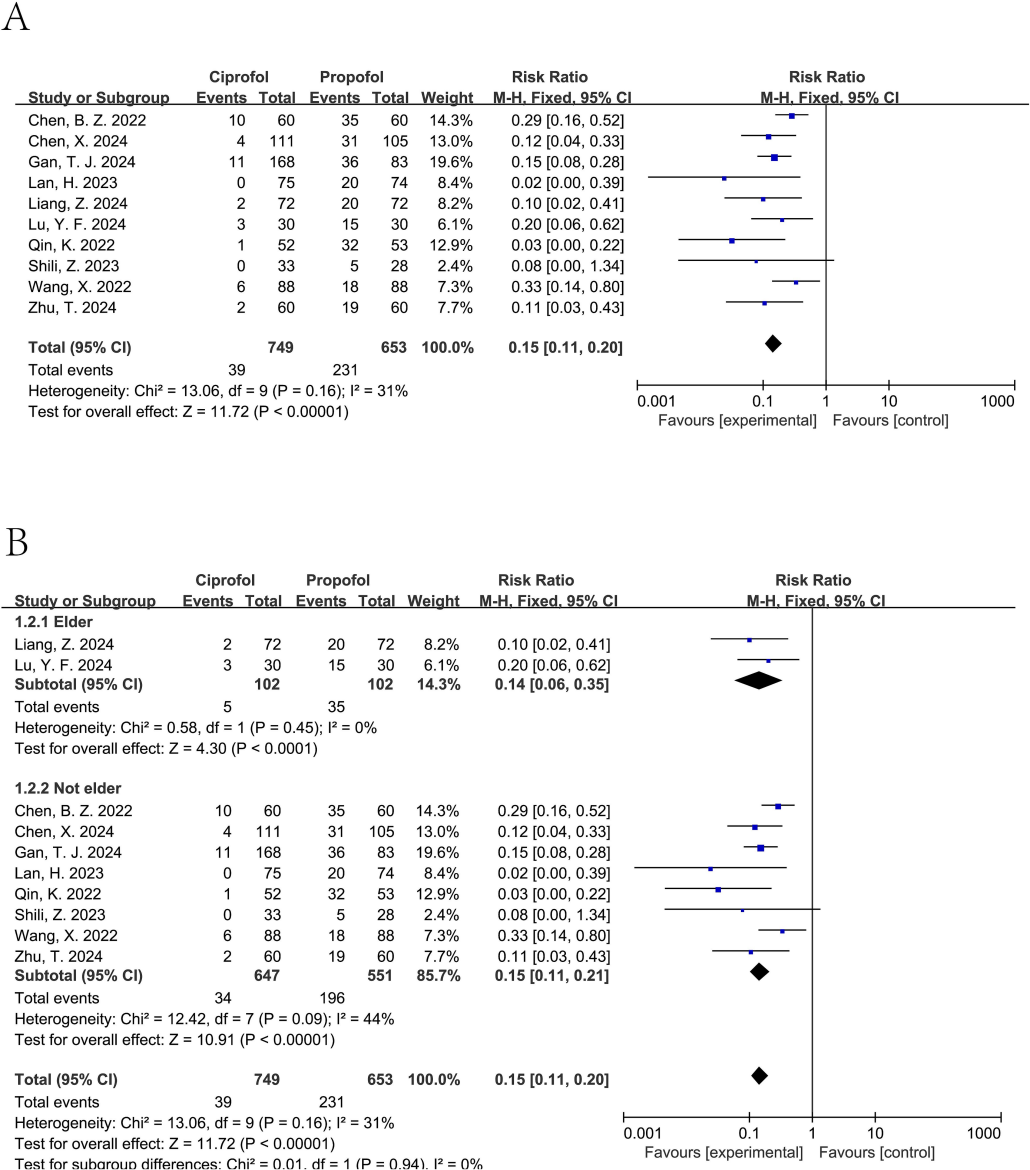
Forest plot of injection pain comparing ciprofol and propofol. **(A)** Overall analysis using a fixed-effect model. **(B)** Subgroup analysis by age (elderly vs. non-elderly). *I*^2^ values indicate between-study heterogeneity.

**Figure 4:**
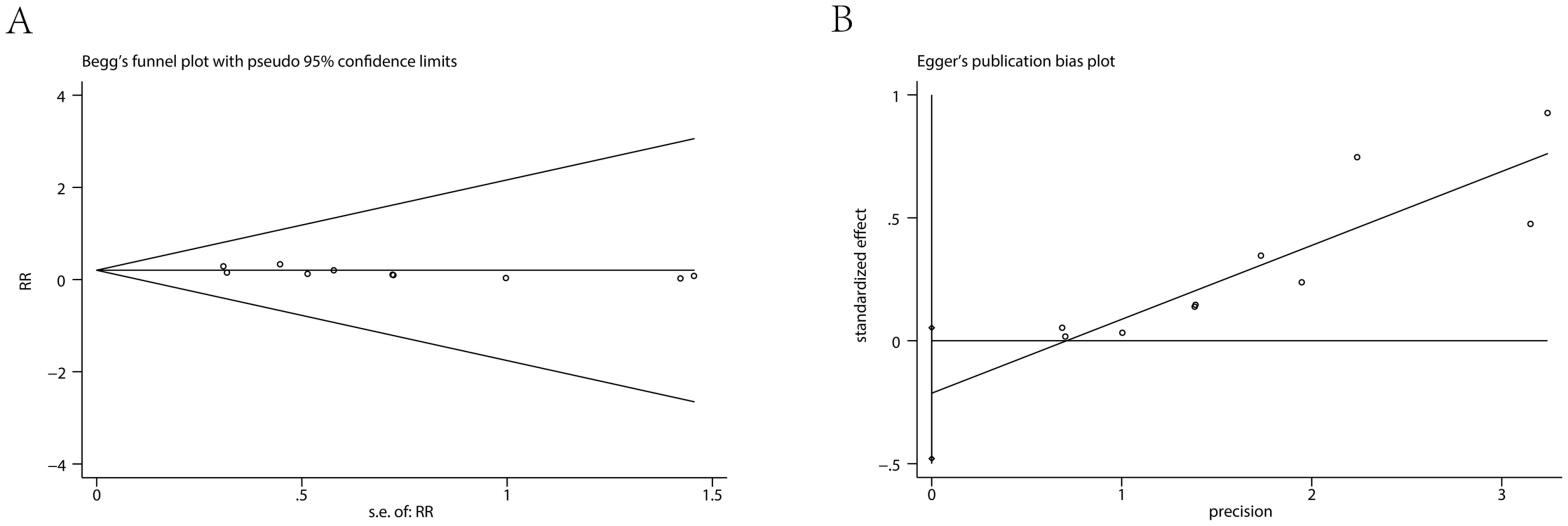
Results of the Begg’s test **(A)** and Egger’s test **(B)**.

An examination of ten separate clinical studies (11–13, 15–21), encompassing a total of 1,402 subjects, compared the sensation of pain when injected with either ciprofol or propofol. Findings from the synthesis of these trials demonstrate that those administered ciprofol experienced substantially less pain at the injection site. The compiled data indicate a markedly reduced risk of pain for the ciprofol cohort, as evidenced by a pooled relative risk of 0.15 (95% confidence interval: 0.11 to 0.20, *I*^2^ = 31%), signifying a distinct benefit in favor of ciprofol (Figure 3A). Analysis of publication bias employing Begg’s (*p* = 0.655) and Egger’s (*p* = 0.102) methodologies revealed no considerable bias when assessing the injection discomfort associated with the two substances (Figure 4). Additional subgroup evaluations were performed to investigate elements affecting the pain experienced during injection and to clarify the relative effectiveness of ciprofol compared to propofol. Key procedural characteristics related to injection pain varied across studies and are detailed in Supplementary Table S2.

#### 3.5.2 Patient age

Ciprofol was associated with a significant reduction in injection pain for elderly patients (pooled RR from 2 trials (17, 18): 0.14, 95% CI: 0.06 to 0.35, *I*^2^ = 0%), as well as for non-elderly patients (pooled RR from 8 trials (11–13, 15, 16, 19–21): 0.15, 95% CI: 0.11 to 0.21, *I*^2^ = 44%) (see Figure 3B).

#### 3.5.3 The incidence of hypotension, hypertension, bradycardia, tachycardia, and PONV

**Figure 5:**
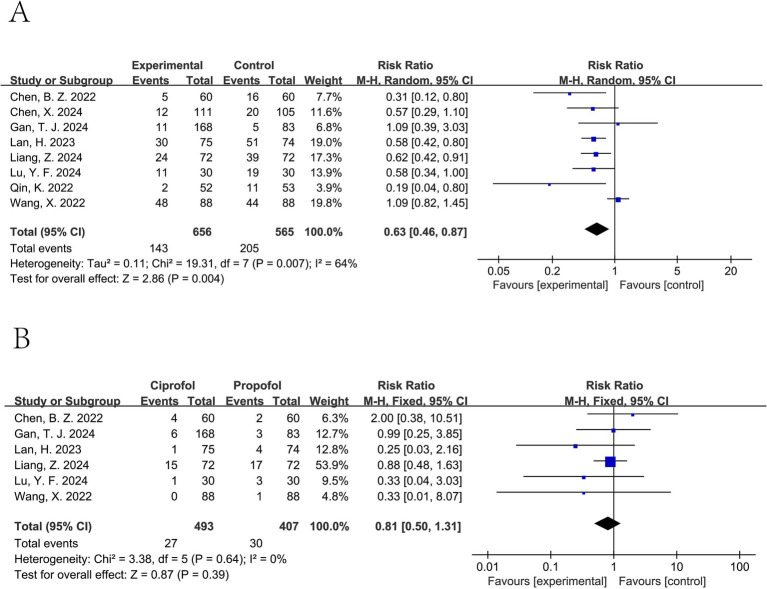
Forest plot of hypotension **(A)** and hypertension **(B)**. A random-effects model was used for hypotension due to substantial heterogeneity (*I*^2^ > 50%), and a fixed-effect model was used for hypertension.

**Figure 6:**
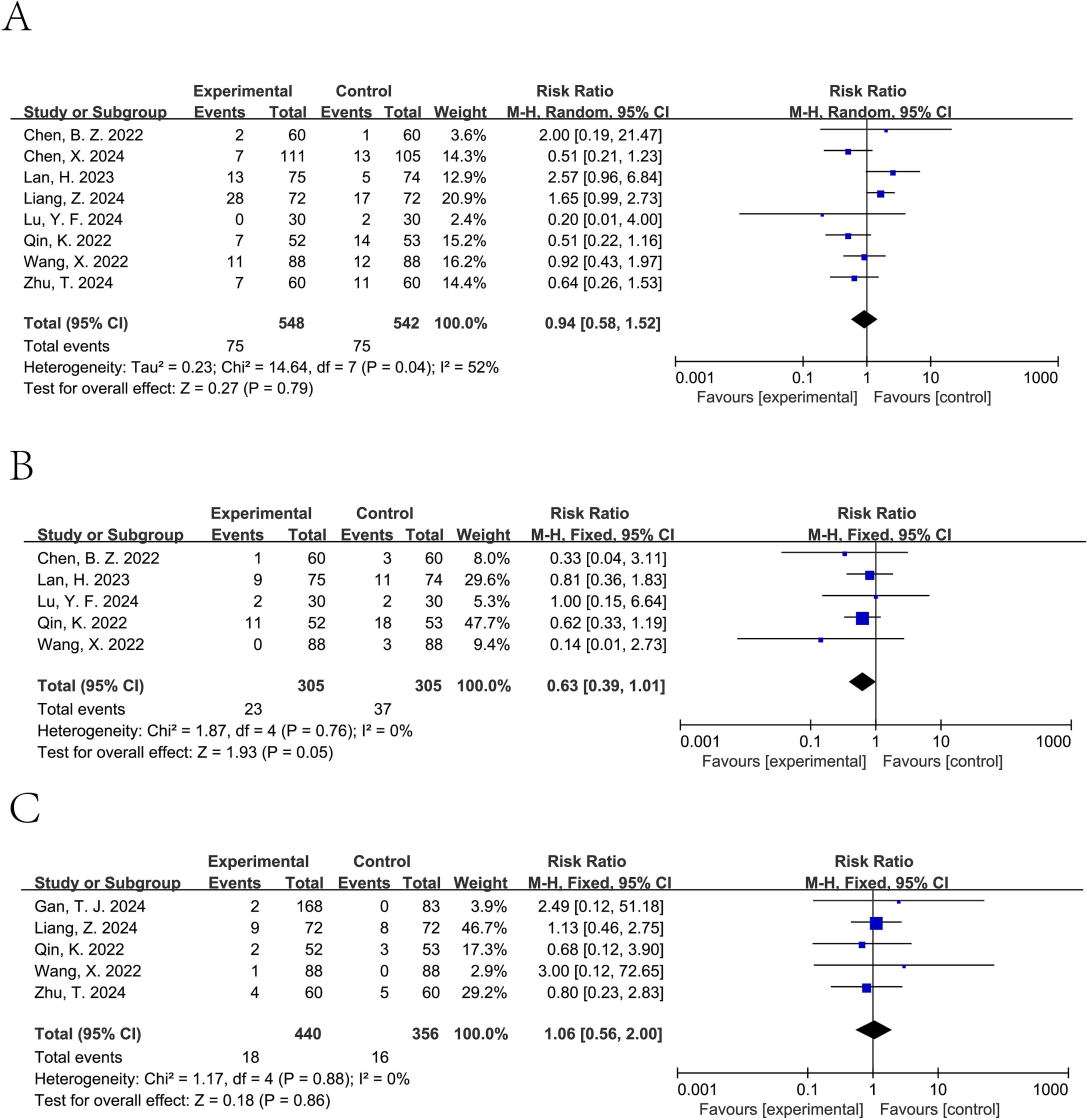
Forest plot of bradycardia **(A)**, tachycardia **(B)**, and PONV **(C)**. Random-effects models were used when *I*^2^ > 50%.

Ciprofol significantly decreased the incidence of hypotension (pooled RR from 8 studies involving a total of 1,221 patients (11–13, 15–18, 20): 0.63, 95% CI: 0.46 to 0.87, *I*^2^ = 64%) (Figure 5A). For tachycardia, a lower incidence was observed in the ciprofol group; however, this difference was not statistically significant (pooled RR from 5 studies involving a total of 610 patients (12, 13, 15, 18, 20): 0.63, 95% CI: 0.39 to 1.01, *I*^2^ = 0%) (Figure 6B). It did not significantly affect the rates of hypertension (pooled RR from 6 studies involving a total of 900 patients (12, 15–18, 20): 0.81, 95% CI: 0.50 to 1.31, *I*^2^ = 0%) (Figure 5B), bradycardia (pooled RR from 8 studies involving a total of 1,090 patients (11–13, 15, 17, 18, 20, 21): 0.94, 95% CI: 0.58 to 1.52, *I*^2^ = 52%) (Figure 6A), or PONV (pooled RR from 5 studies involving a total of 796 patients (13, 16, 17, 20, 21): 1.06, 95% CI: 0.56 to 2.00, *I*^2^ = 0%) (Figure 6C).

### 3.6 Trial sequential analysis

**Figure 7:**
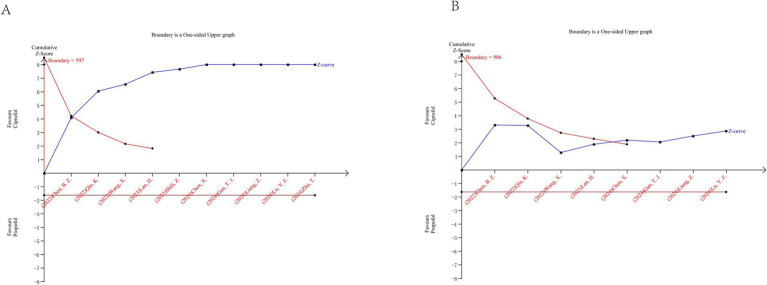
Trial sequential analysis results. **(A)** Injection pain; **(B)** Hypotension.

Trial sequential analysis was conducted for injection pain and hypotension. For injection pain, TSA included 10 randomized controlled trials involving a total of 1,402 patients. The cumulative Z-curve crossed the trial sequential monitoring boundary for benefit before reaching the required information size, indicating that the available evidence was sufficient to confirm a reduction in injection pain with ciprofol compared with propofol (Figure 7A). For hypotension, TSA included 8 randomized controlled trials with a total of 1,221 patients. Similarly, the cumulative Z-curve crossed the monitoring boundary for benefit before reaching the required information size, suggesting that the current evidence was sufficient to support a lower incidence of hypotension associated with ciprofol (Figure 7B).

Overall, TSA findings indicate that further trials are unlikely to change the conclusions for these two outcomes.

## 4 Discussion

Administering ciprofol intravenously, as with propofol, frequently results in pain at the injection site (22). This meta-analysis was conducted to investigate the incidence of injection pain following anesthesia with ciprofol in comparison to propofol. The findings yielded several significant insights. To begin with, patients experienced significantly less discomfort from injections when treated with ciprofol compared to those who received propofol, indicating that ciprofol may offer advantages over propofol with respect to injection pain during the induction of general anesthesia, based on the outcomes assessed in the included trials. Secondly, ciprofol was effective in reducing injection pain across various age groups, providing relief from discomfort in both elderly and younger patients when compared to propofol. Furthermore, although ciprofol significantly lowered the incidence of hypotension, while a non-significant trend toward lower tachycardia was observed, it did not lead to notable changes in the rates of hypertension, bradycardia, or PONV, which are commonly observed cardiovascular side effects. These results indicate that ciprofol not only enhances the patient experience by minimizing injection pain but also presents some cardiovascular benefits, suggesting that ciprofol may be a reasonable alternative to propofol for anesthesia induction, particularly in terms of injection pain and selected hemodynamic outcomes.

Several recent meta-analyses have compared ciprofol with propofol for anesthesia induction or procedural sedation; however, most focused on overall efficacy and safety rather than injection pain specifically. Our review differs from previous analyses by using injection pain as the primary outcome and by restricting inclusion to randomized controlled trials in patients. This provides a more clinically focused and methodologically rigorous assessment of this important patient-centered outcome.

Moderate heterogeneity was observed for injection pain (*I*^2^ = 31%) and non-elderly subgroups (*I*^2^ = 44%), while substantial heterogeneity was present for hypotension (*I*^2^ = 64%) and bradycardia (*I*^2^ = 52%). These differences are likely explained by variations in surgical type, anesthetic dosing, maintenance regimens, and peri-induction management across trials. In particular, differences in injection rate, cannula size, venous site, and premedication are known to influence injection pain but were not uniformly reported, which may have contributed to between-study variability.

Trial sequential analysis strengthens the reliability of our findings by accounting for the risk of random errors due to sparse data and repeated testing. The fact that the cumulative Z-curves for both injection pain and hypotension crossed the trial sequential monitoring boundaries before reaching the required information size indicates that the observed effects are unlikely to be due to chance and that further trials are unlikely to materially change these conclusions.

Injection pain can cause immediate discomfort, leading to muscle tension, bruising, and even vasovagal responses, while repeated pain may trigger inflammation or infection (23). Psychologically, frequent injection pain can lead to needle anxiety or even phobia, discouraging patients from necessary treatments and impacting health outcomes (24). Pain also activates the stress response, raising blood pressure and heart rate, which can be risky for cardiovascular patients and may hinder healing (25). Additionally, injection pain often reduces treatment adherence in therapies requiring frequent doses. Injection pain associated with propofol presents a significant drawback to its use in anesthesia (26). The discomfort experienced during a propofol injection primarily stems from the irritation of the blood vessel linings (27). This irritation triggers the activation of pain receptors located within the venous structures, resulting in a heightened sensation of pain during the injection process (28). This activation transmits pain signals to the brain, causing discomfort during administration. This pain can be attributed to several factors related to propofol’s formulation and administration. The lipid emulsion used to deliver propofol may cause irritation to adjacent tissues, and the rate of injection is a crucial factor, faster injections typically increase discomfort (17). Additionally, propofol’s acidic pH can exacerbate pain at the injection site, contributing to a less comfortable experience for patients (29). Conversely, the molecular composition and specific formulation of ciprofol are linked to a lower incidence of pain upon injection (30). Although chemically similar to propofol, ciprofol features modifications that likely lead to decreased activation of pain receptors in the vascular walls, resulting in less tissue irritation (31). The lipid emulsion of ciprofol may consist of smaller or more stable particles, which can minimize vascular inflammation and irritation during injection (32). Ciprofol possesses a lower acidic pH compared to propofol, which contributes to a decrease in injection site discomfort (33). Ciprofol is approximately five times more potent than propofol, requiring only about 20% of the typical dose to achieve equivalent anesthetic effects, which could lead to reduced drug volumes, lower side effect risks, and improved patient comfort during anesthesia (18). This reduced dosage results in lower exposure of vascular tissues to potential irritants, contributing to the overall decrease in injection pain. While the current evidence suggests that ciprofol provides a more comfortable injection experience, further research is necessary to elucidate the precise mechanisms underlying these differences in injection pain between ciprofol and propofol.

Ciprofol is less likely to cause hypotension compared to propofol due to its favorable pharmacological profile, allowing effective sedation at lower doses, which minimizes cardiovascular impact (34). The quick initiation and short-lasting effects of anesthesia are vital for preserving stable blood circulation during a range of medical interventions (35). Additionally, ciprofol’s greater affinity for GABA receptors provides sedation without excessive autonomic suppression, further contributing to cardiovascular stability. In contrast, propofol reduces intracellular calcium concentration, leading to decreased myocardial contraction and inhibiting vascular tone, which can cause hypotension (36). Both agents can induce transient hypotension through mechanisms like peripheral vasodilation and reduced myocardial contractility. Ciprofol has a lower incidence of tachycardia compared to propofol due to its ability to achieve effective sedation at lower doses, minimizing sympathetic nervous system stimulation (37). Its favorable hemodynamic profile promotes cardiovascular stability without excessive autonomic suppression, reducing the risk of reflex tachycardia (38). Additionally, ciprofol’s formulation may cause less vascular irritation, contributing to its lower likelihood of inducing tachycardia (39).

Limitations and suggestions for practice: The studies included in this analysis exhibit several limitations, underscoring the necessity for additional research. First, although 10 randomized controlled trials were included, the sample size for several secondary outcomes remained limited, which may reduce statistical power. Second, some domains of risk of bias, particularly allocation concealment and blinding, were judged as unclear in several trials, which may have influenced the pooled estimates. Third, injection pain is highly sensitive to procedural factors such as injection site, injection rate, cannula characteristics, and pre-treatment. However, these details were not consistently reported across the included trials. To improve transparency, we summarized all available study-level procedural information in Supplementary Table S2, with unreported items clearly labeled as “not specified.” This incomplete reporting may have contributed to clinical heterogeneity and should be addressed in future trials. Finally, most included studies were conducted in China, which may limit the generalizability of the findings to other populations.

## 5 Conclusion and recommendations

Based on the available randomized controlled trials, ciprofol was associated with a significantly lower incidence of injection pain during the induction of general anesthesia compared with propofol, and was also associated with a reduced incidence of hypotension, while a trend toward lower tachycardia was observed. Trial sequential analysis supported the robustness of the findings for injection pain and hypotension, suggesting that further trials are unlikely to alter these conclusions. Within the context of anesthesia induction and the outcomes assessed, ciprofol appears to be a promising alternative to propofol. However, additional high-quality studies are warranted to further evaluate its safety and efficacy in broader clinical settings and other anesthetic contexts.

## Data Availability

The original contributions presented in the study are included in the article/Supplementary material, further inquiries can be directed to the corresponding author.
