# Supplementary material for: Incidence of injection pain between ciprofol and propofol after induction of general anesthesia: a systematic review and meta-analysis of randomized controlled trials
Source: Front Med (Lausanne). 2026 Feb 3;13:1749156. doi: 10.3389/fmed.2026.1749156 (PMC12909516; doi:10.3389/fmed.2026.1749156)
Supplement: Supplementary file 1 [file Table_1.docx]

**Full Electronic Search Strategies**

Objective

To identify all randomized controlled trials comparing ciprofol and propofol with respect to injection pain and related adverse events during the induction of general anesthesia.

Databases searched

PubMed (MEDLINE)

Embase (via Elsevier)

Cochrane Central Register of Controlled Trials (CENTRAL)

Search period

From database inception to 29 July 2025

Search limits

No language restrictions

Human studies

Randomized controlled trials (RCTs) were included during the study selection stage

**Search concepts**

The search strategy combined the following core concepts:

Ciprofol / HSK3486

Propofol

Injection pain / vascular pain / pain on injection

These concepts were combined using Boolean operators (AND / OR) as detailed below.

**1. PubMed (MEDLINE)**

Search date: 29 July 2025

Search strategy:

Ciprofol terms

ciprofol[Title/Abstract] OR

HSK3486[Title/Abstract] OR

"HSK 3486"[Title/Abstract]

Propofol terms

"Propofol"[MeSH Terms] OR

propofol[Title/Abstract]

Injection pain terms

"Pain"[MeSH Terms] OR

pain[Title/Abstract] OR

"injection pain"[Title/Abstract] OR

"injection site pain"[Title/Abstract] OR

"pain on injection"[Title/Abstract] OR

"vascular pain"[Title/Abstract]

Combined search

#1 AND #2 AND #3

Final PubMed query:

((ciprofol[Title/Abstract] OR HSK3486[Title/Abstract] OR "HSK 3486"[Title/Abstract])

AND("Propofol"[MeSH Terms] OR propofol[Title/Abstract])AND("Pain"[MeSH Terms] OR pain[Title/Abstract] OR "injection pain"[Title/Abstract]OR "injection site pain"[Title/Abstract] OR"pain on injection"[Title/Abstract]OR "vascular pain"[Title/Abstract]))

Date limit: inception to 29 July 2025

**2. Embase (via Elsevier)**

Search date: 29 July 2025

Search strategy (Emtree + free-text terms):

Ciprofol terms

'ciprofol'/exp OR

ciprofol:ti,ab OR

hsk3486:ti,ab OR

'hsk 3486':ti,ab

Propofol terms

'propofol'/exp OR

propofol:ti,ab

Injection pain terms

'injection pain'/exp OR

'pain'/exp OR

'injection pain':ti,ab OR

'injection site pain':ti,ab OR

'pain on injection':ti,ab OR

'vascular pain':ti,ab

Study design terms (RCT filter)

'randomized controlled trial'/exp OR

random*:ti,ab OR

placebo*:ti,ab OR

'double blind':ti,ab OR

'single blind':ti,ab

Combined search

#1 AND #2 AND #3 AND #4

Limits applied:

Humans

No language restriction

Date limit: inception to 29 July 2025

**3. Cochrane Central Register of Controlled Trials (CENTRAL)**

Search date: 29 July 2025

Search strategy:

(ciprofol OR HSK3486 OR "HSK 3486")
AND

(propofol)
AND

("injection pain" OR "pain on injection" OR "injection site pain" OR "vascular pain" OR pain)

Search fields: Title, Abstract, Keywords

Date limit: inception to 29 July 2025
