# Supplementary material for: Incidence of injection pain between ciprofol and propofol after induction of general anesthesia: a systematic review and meta-analysis of randomized controlled trials
Source: Front Med (Lausanne). 2026 Feb 3;13:1749156. doi: 10.3389/fmed.2026.1749156 (PMC12909516; doi:10.3389/fmed.2026.1749156)
Supplement: Supplementary file 2 [file Table_2.docx]

Supplementary Table S2. Study-level procedural characteristics related to injection pain assessment

| Study (Year) | Injection site | Cannula size | Injection rate | Pre-treatment (e.g., lidocaine/opioids) | Timing of pain assessment | Pain assessment method |
| --- | --- | --- | --- | --- | --- | --- |
| Shili, Z.et al. (2023) | Not specified | Not specified | Not specified | None (No pre-treatment mentioned for injection pain prevention) | During injection (intraoperative) | Clinical observation (recording incidence of injection pain as adverse event) |
| Zhu et al. (2024) | Not specified | Not specified | Induction: Ciprofol (0.4 mg/kg), Propofol (2.0 mg/kg); Maintenance: Ciprofol (initial 0.8 mg·kg⁻¹·h⁻¹, range 0.3-2.4 mg·kg⁻¹·h⁻¹), Propofol (initial 5.0 mg·kg⁻¹·h⁻¹, range 3-12 mg·kg⁻¹·h⁻¹) | Ringer’s solution (200-300 mL) before induction; sufentanil (0.4 μg/kg) and rocuronium (0.9 mg/kg) after study drug administration | During anesthesia induction | Clinical observation (recording incidence of injection pain as adverse event) |
| Gan et al. (2023) | Dorsum of hand, antecubital fossa, forearm | Not specified | Ciprofol (0.4 mg/kg IV slow injection over 30±5 sec, top-up 0.2 mg/kg if needed); Propofol (2.0 mg/kg IV slow injection over 30±5 sec, top-up 1.0 mg/kg if needed) | Fentanyl (1 μg/kg, max 100 μg) within 5 minutes prior to study drug; no lidocaine administered | During initial administration of study drug (prior to loss of consciousness) and after transfer to PACU | Numerical Rating Scale (NRS, score ≥1 defined as injection-site pain) |
| Qin et al. (2022) | Not specified | Not specified | Induction: Ciprofol (0.4 mg/kg, 10-30 s), Propofol (2.0 mg/kg); Maintenance: Ciprofol (0.8-2.4 mg·kg⁻¹·h⁻¹), Propofol (4-12 mg·kg⁻¹·h⁻¹) | Sufentanil (0.4-0.5 μg/kg) and cisatracurium (0.2 mg/kg) after induction drug administration; no pre-treatment for injection pain | During anesthesia induction | Clinical observation (recording incidence of injection pain as adverse event) |
| Wang et al. (2022) | Not specified | Not specified | Ciprofol (0.4 mg/kg IV bolus over 30 s, top-up 0.2 mg/kg if needed); Propofol (2.0 mg/kg IV bolus over 30 s, top-up 1.0 mg/kg if needed) | Midazolam (0.04 mg/kg) and sufentanil (0.3 μg/kg) 2 min prior to study drug; no lidocaine administered | During injection of study drug | Clinical observation (recording incidence of injection pain as adverse event) |
| Lan et al. (2023) | Upper extremity vein | Not specified | Induction: Ciprofol (0.4 mg/kg over 30 s), Propofol (2.0 mg/kg over 30 s); Maintenance: Ciprofol (0.6-1.2 mg·kg⁻¹·h⁻¹), Propofol (3.0-6.0 mg·kg⁻¹·h⁻¹) | Sufentanil (0.1 μg/kg) prior to sedative administration; no lidocaine administered | After the first injection of study drug | Verbal report by patients (recording incidence of injection pain as adverse event) |
| Lu et al. (2024) | Hand vein | Not specified | Ciprofol (0.3 mg/kg IV injection over 30 s); Propofol (1.5 mg/kg IV injection over 30 s) | No premedication; sufentanil (0.3-0.4 μg/kg) and cisatracurium (0.15 mg/kg) after loss of consciousness | During drug injection | Verbal inquiry + Numerical Rating Scale (NRS ≥3 defined as injection pain) |
| Liang et al. (2024) | Not specified | Not specified | Induction: Ciprofol (0.2-0.5 mg/kg IV), Propofol (1-2 mg/kg IV); Maintenance: Ciprofol (0.4-3 mg·kg⁻¹·h⁻¹), Propofol (4-12 mg·kg⁻¹·h⁻¹) | No premedication; alfentanil (40 μg/kg) and rocuronium (1 mg/kg) after induction drug administration | During anesthesia induction | Four-point scale by Ambesh et al. (recording incidence of injection pain as adverse event) |
| Chen B.Z. et al. (2022) | Dorsum of the right hand | 18-Gauge intravenous cannula | Ciprofol (0.4 mg/kg manual injection over 30 s); Propofol (2 mg/kg manual injection over 30 s) | Midazolam (0.03 mg/kg) and sufentanil (0.3 μg/kg) 2 min prior to study drug; no lidocaine administered | During study drug injection until successful induction (MOAA/S ≤1) | Verbal inquiry + Numerical Rating Scale (NRS ≥3 or withdrawal response defined as injection pain) |
| Chen.X. et al. (2024) | Not specified | Not specified | Ciprofol (0.4 mg/kg IV slow injection over 30-40 s); Propofol (2.0 mg/kg IV slow injection over 30-40 s) | Midazolam (0.04 mg/kg) and sufentanil (0.3 μg/kg) prior to induction drug administration; no lidocaine administered | During anesthesia induction | Clinical observation (recording incidence of injection pain as adverse event) |

Abbreviations: NRS, Numerical Rating Scale; NR, not reported.
Note: “Not specified” indicates that the information was not reported in the original publication.
